# EPR Oximetry Sensor—Developing a TAM Derivative for In Vivo Studies

**DOI:** 10.1007/s12013-017-0824-3

**Published:** 2017-09-04

**Authors:** Agnieszka Boś-Liedke, Magdalena Walawender, Anna Woźniak, Dorota Flak, Jacek Gapiński, Stefan Jurga, Małgorzata Kucińska, Adam Plewiński, Marek Murias, Marwa Elewa, Lisa Lampp, Peter Imming, Krzysztof Tadyszak

**Affiliations:** 1 NanoBioMedical Centre, Adam Mickiewicz University, ul. Umultowska 85, 61614 Poznań, Poland; 2 Faculty of Physics, Adam Mickiewicz University, ul. Umultowska 14, 61614 Poznań, Poland; 3 Department of Toxicology, Poznan University of Medical Sciences, ul. Dojazd 30, 60631 Poznan, Poland; 4 Faculty of Pharmacy, Suez Canal University, P.O. 41522, Ismailia, Egypt; 5 Institute of Pharmacy, Martin Luther University Halle-Wittenberg, Wolfgang-Langenbeck-Str. 4, 06120 Halle (Saale), Germany; 6 Institute of Molecular Physics, Polish Academy of Sciences, ul. M. Smoluchowskiego 17, 60179 Poznań, Poland

**Keywords:** EPR oximetry, TAM derivative, Oxygenation sensor, Cytotoxicity

## Abstract

Oxygenation is one of the most important physiological parameters of biological systems. Low oxygen concentration (hypoxia) is associated with various pathophysiological processes in different organs. Hypoxia is of special importance in tumor therapy, causing poor response to treatment. Triaryl methyl (TAM) derivative radicals are commonly used in electron paramagnetic resonance (EPR) as sensors for quantitative spatial tissue oxygen mapping. They are also known as magnetic resonance imaging (MRI) contrast agents and fluorescence imaging compounds. We report the properties of the TAM radical tris(2,3,5,6-tetrachloro-4-carboxy-phenyl)methyl, (PTMTC), a potential multimodal (EPR/fluorescence) marker. PTMTC was spectrally analyzed using EPR and characterized by estimation of its sensitivity to the oxygen in liquid environment suitable for intravenous injection (1 mM PBS, pH = 7.4). Further, fluorescent emission of the radical was measured using the same solvent and its quantum yield was estimated. An in vitro cytotoxicity examination was conducted in two cancer cell lines, HT-29 (colorectal adenocarcinoma) and FaDu (squamous cell carcinoma) and followed by uptake studies. The stability of the radical in different solutions (PBS pH = 7.4, cell media used for HT-29 and FaDu cells culturing and cytotoxicity procedure, full rat blood and blood plasma) was determined. Finally, a primary toxicity test of PTMTC was carried out in mice. Results of spectral studies confirmed the multimodal properties of PTMTC. PTMTC was demonstrated to be not absorbed by cancer cells and did not interfere with luciferin-luciferase based assays. Also in vitro and in vivo tests showed that it was non-toxic and can be freely administrated till doses of 250 mg/kg BW via both i.v. and i.p. injections. This work illustrated that PTMTC is a perfect candidate for multimodal (EPR/fluorescence) contrast agent in preclinical studies.

## Introduction

The concentration of oxygen in tissues is one of the most crucial physiological parameters [1]. In most healthy tissues, the oxygen partial pressure (pO_2_) ranges between 10 and 80 mmHg. Low oxygen concentration (hypoxia) is associated with various pathophysiological processes in different organs, e.g., stroke or cancer. In solid tumors, fast angiogenesis of the cancer cells stimulates the creation of chaotic and poorly organized vasculature that in turn results in deprivation of both oxygen (pO_2_ < 5 mmHg) and nutrients supply to the tissue that further stimulates even faster angiogenesis of the pathological cells [2]. In those tissues hypoxia is of special importance causing a poor response of the tumor to radio-, chemo- or even surgical treatment [3, 4]. Resistivity of the cancer tissues due to low oxygen content is explained by limited interaction of the oxygen with radicals formed by radiation. Those products cause DNA damages that cannot be easily repaired [5]. Furthermore, hypoxia increases also a predisposition to metastasis and is associated with poor prognosis and survival rate of the patients [6].

The first hypoxia studies appeared in the nineteenth century and were closely related to the recently developed discipline of radiation oncology—a combination of medicine, physics, chemistry, engineering and biology. At the same time it became clear that improvement of radiotherapeutical medical care is closely related to its quantitative planning, careful documentation, determination of toxicity, and benefits for patients, as well as development of new imaging techniques and technology in this field. This fact became a motivation for analyzing physiological parameters, like perfusion or oxygenation of the cancer tissue that change, upon treatment and have an influence on its efficiency. The quantitative measurement of those parameters during applied radiochemotherapy would significantly help in prediction of therapy success and evaluation of its effectiveness [7]. Therefore, the quantification of such parameters, monitoring of their changes and imaging of distribution in time became mandatory in radiation oncology.

Until today, methods for hypoxia imaging in human cancers have been accomplished with highly sensitive but low resolution methods presented by nuclear medicine, i.e., ^18^F-fluoromisonidazole positron emission tomography (PET) (F-18-FMISO-PET); and with qualitative radiological methods, e.g., blood oxygen level-dependent magnetic resonance imaging (BOLD-MRI), dynamic contrast-enhance MRI (DCE-MRI), Overhauser MRI (OMRI), contrast enhanced computer tomography, electron paramagnetic resonance imaging, and ^19^F oximetry [8–13]. Only some of them allow not only for qualitative, but also for quantitative evaluation of tissue oxygenation. One of the best examples and widely used in clinics is F-18 FMISO PET. This technique uses an intravenously injected dose of radioactive isotope (F-18) as a signal source that is targeted by metabolism of nitroimidazole in cells [14]. Another method used for quantitative imaging is electron paramagnetic resonance (EPR) oxymetry. It delivers not only spectroscopic information about the partial pressure of the oxygen deposited in the tissues, but also allows the creation of 3D and 4D tomographic images. The amount of paramagnetic molecular oxygen present in biological systems is indicated by the linewidth broadening of the intravenously (i.v.) or intraperitoneal (i.p.) injected soluble paramagnetic spin probe. The broadening results from Heisenberg exchange between molecular O_2_ dissolved in the blood and the radical [15]. Although the material is also injected into the blood stream, it is not radioactive, so the delivery to the patient does not requires special procedures, protection and trainings of the staff, longer hospitalization of the patients that are exposed to the radiation. In contrast to radionuclide probes, the spin probes can be stored for a long time and need not be produced just before use. Among soluble spin probes, two types are of special interest for diagnostic purposes, namely nitroxides and triarylmethyl (TAM) radicals. Although nitroxides present a number of different properties that can be manipulated, the TAM radicals are particularly convenient for in vivo imaging application because of their very narrow, single EPR lines. Line widths are less than 100 mGauss and linearly dependent on the oxygen concentration with high sensitivity [16].

TAM radicals also found an application in MRI [11, 17] by use of the Overhauser effect. Furthermore, some of them are photosensitive and capable to fluorescence. This property has never been deeply studied in preclinical and clinical aspects. According to our knowledge, the only research in this line was published in 1944 by Lewis, Lipkin, and Magel under the title “The Light Absorption and Fluorescence of Triarylmethyl Free Radicals” [18]. All of the mentioned properties make TAM radicals suitable candidates for multimodal imaging contrast agents.

Due to the importance of the preclinical and clinical significance of tissue oxygenation estimation and imaging, an increasing number of EPR TAM radicals are being evaluated. This study reports on EPR oxygen sensor based on TAM derivative, which is a potential candidate for an efficient multimodal imaging. As a potential in vivo hypoxia marker for EPR imaging, the PTMTC TAM radical was firstly characterized towards its sensitivity to the oxygen in liquid media suitable for intravenous injection (1 mM PBS, pH = 7.4). Then the fluorescence of the radical in PBS was measured and its quantum yield was determined. Cytotoxicity was examined using two cell lines, HT-29 (colorectal adenocarcinoma, less hypoxic) and FaDu (squamous cell carcinoma, more hypoxic). Also the stability of the radical in different solutions (PBS pH = 7.4, cell media used for HT-29 and FaDu cell culturing and cytotoxicity procedure, full rat blood, and blood plasma) was determined. The cytotoxicity test was followed by additional studies focused on PTMTC uptake by cancer cells and its interference with luciferin–luciferase-based assays. Finally, PTMTC was administrated to the Balb/c mice at the dose of 250 mg/kg BW via i.v. and i.p. injections to estimate its in vivo toxicity.

## Materials and Methods

### Synthesis

#### Tris(4-carboxy-2,3,5,6-tetrachloro-phenyl)methyl radical (PTMTC)

Note: When the radicals are in solution, light should be excluded.

Tris(4-ethoxy-carbonyl-2,3,5,6-tetrachlorophenyl)methyl radical [19, 20] (500 mg, 0.58 mmol) was suspended in conc. sulfuric acid (95%, 60 mL). The mixture was kept at 90 °C for 12 h. The resulting solution was cooled and poured very slowly onto ice. The aqueous phase was extracted twice with 150 mL each of tert-butyl methyl ether. The organic phases were combined and concentrated to approx. 100 mL and extracted with aqueous 10% sodium carbonate. The carbonate phase was acidified slowly with 5 M hydrochloric acid and extracted four times with 50 mL each of tert-butyl methyl ether. The organic phases were combined, dried over anhydrous sodium sulfate, filtered, and the solvent removed under vacuum. The residue was dissolved in diethyl ether (approx. 10 mL) and the product precipitated by slow addition of n-heptane. This purification step was repeated three times. Yield: approx. 380 mg (84%) of a red powder. Mp. >280 °C. Infrared (IR) (KBr): *ν* = 3702–2643, 1703, and 1661 cm^−1^. MS (ESI): *m*/*z* 788.83 [M]^−^ (70%), 743.60 [M − CO_2_]^−^ (100%), 698.82 [M − 2CO_2_]^−^ (60%). HRMS (ESI): calcd. for C_22_H_4_Cl_12_O_6_ [M + H]^+^ 789.618; found 789.618.

### EPR Spectroscopy

The L-band EPR measurements were carried out using E540 L-Band Bruker^®^ Spectrometer equipped with E 540R23 L-Band EPR-resonator.

Stability measurements in various media were done with simultaneous record of the 7,7,8,8-tetracyanoquinodimethane anion radical (TCNQ) spectrum. The spectral displacement was achieved by applying magnetic field gradient 5 G/cm. The radical amplitudes were measured and normalized by the standard amplitude. The line width measurements were performed with 10,000 points resolution in the field of 2 G around the maximum of the radical absorption signal. Automatic fine tuning after each scan (10 scans, 200 s each) was enabled. Microwave power was 3.6 mW, frequency 1.09 GHz, modulation amplitude 0.01 G, modulation frequency 10 kHz. No saturation effects were visible.

The X-band EPR relaxation measurements were performed with *Bruker ESP 380* FT/EPR X-band spectrometer in room temperature (23 ^o^C). To estimate the spin–spin (T_2*_) and spin–lattice (T_1_) relaxation times, free induction decay (FID) sequence was used. Parameters used for T_2_* assessment were as followed: pulse (*p*_1_) of 24 ns, delay time (*d*_0_) of 96 ns and acquisition in steps of 8 ns. Recorded number of points was 128. Parameters for T_1_ assessment from FID were *p*_1_ = 80 ns, *d*_1_ = 96 ns, and the sequence was (*p*_1_ *d*_1_)_8*x*_ dx (8 ns) *p*_0_ (24 ns) *d*_0_ (104 ns) and acquisition in steps of 8 ns [21]. Number of points recorded 256. Attenuation of microwave pulses was set to 5 dB of the maximal TWT amplifier power. No saturation effects were visible at this power level.

### Sensitivity to Oxygen in Liquid Environment

Simultaneously with the nitrogen gas saturation experiments of the PTMTC (C_22_H_3_Cl_12_O_6_^•^, 788.69 g/M) radical, the EPR spectrum was recorded. The radical was mixed with PBS (pH = 7.4) solution gaining 1 mM of final concentration. The PBS composition was: NaCl 8 g/L, KCl 0.2 g/L, Na_2_HPO_4_ 1.42 g/L, KH_2_PO_4_ 0.24 g/L, and HCl (29 mM/L) for pH adjustment. The solution was boiled, and cooled with simultaneous flushing with N_2_ multiple times. The EPR experiment started when the PreSense® oximeter with NTH-PSt7-02 oxygen microsensor, submerged in the solution, showed diode saturation value. Salinity correction was applied. The vial was open allowing the mixing of air with the solution. The oximeter measurements were performed simultaneously with the EPR measurements. Oxygen gas used in experiments was of 99.999% purity grade.

### Stability of the Radical in Different Solutions

The PTMTC was dissolved in multiple solvents (always 1 mM of radical concentration):

1. Pure PBS buffer (pH = 7.4).
2. Eagle’s minimum essential medium (EMEM) for cell culture (1 mL) supplemented with 10% of fetal bovine serum (FBS), 1% antibiotic (penicillin 100 µg/mL, streptomycin 100 μg/mL, and P/S) and 250 μL of PBS (pH = 7.4) buffer.
3. McCoy’s cell culture medium (1 mL) supplemented with 10% of FBS, 1% antibiotic (P/S) and 250 μL of PBS (pH = 7.4) buffer.
4. Solution of full rat heart blood (750 μL) mixed with PBS buffer (250 μL, pH = 7.4).
5. Solution of rat blood plasma (750 μL) mixed with PBS buffer (250 μL, pH = 7.4) and stored 4 days in 5 °C before the use.

### Fluorescence Spectroscopy

Fluorescence emission of freshly prepared 1.16 mM PTMTC radical solution samples in PBS (pH = 7.4) was measured in the range of 500–850 nm and at different excitation wavelengths from 320 to 480 nm. The spectra were recorded on the Gilden Photonics FluoroSENS 9000 spectrometer equipped with 150 W continuous Xenon Arc Lamp (Ozone Free, Osram XBO 150 W/CR OFR). As a background reference, the emission of the pure PBS buffer was measured.

### Cytotoxicity Tests

A human colorectal adenocarcinoma cell line (HT-29, ATCC^®^ HTB-38™) and pharynx squamous cell carcinoma (FaDu, ATCC^®^ HTB-43™) were maintained in EMEM (for HT-29 cells) and McCoy’s (for FaDu cells), supplemented with 10% of FBS and 1% of antibiotics (penicillin 100 µg/mL, streptomycin 100 μg/mL) at 37^o^C and 5% CO_2_. For exposure, cells were cultured in 96-well plates. After 24 h cells were exposed to PTMTC solutions in complete medium (2.5, 1.25, and 0.625 mM) for 24, 48, and 72 h. After exposure time, WST-1 assay (*Premix WST-1 Cell Proliferation Assay System*; Takara, Clontech) were performed according to the producers’ procedure. Briefly, 10 μL of Premix WST-1 Cell Proliferation Assay System Reagent were added into each wells, then after 2–4 h the absorbance at wavelength 450 nm were read (ref. wavelength 620 nm).

Cell exposure experiments were carried out in duplicates with positive (without PTMTC) and negative (DMSO) control.

### The Uptake of PTMTC and Interference with Luciferin–Luciferase-Based Assays

The human prostate adeniocarcinoma LNCaP cells were stably transfected with a luciferase reporter gene as previously described [22]. The cells were seeded in 2.5 cm Petri dishes at density 0.5 × 10^6^ cells per dish at allowed to attach overnight. Subsequently the dishes were washed twice with PBS and PTMTC (stock concentration 10 mM in PBS) was added at concentrations 1 mM and incubated for 2 and 6 h. After the incubation the medium was removed, the cells were washed with PBS and fresh medium containing 150 µg/mL luciferine was added to the dishes. The luminescence and fluorescence (excitation at 465 nm, emission at 720 nm) was measured using IVIS Spectrum (Caliper Life Sciences, Hopkington, MA, USA).

### In vitro Confocal Studies

HT-29 cell were seeded into LabTech, after 24 h medium was washed out and PTMTC (concentration of 2.5 mM) was added and incubated for 24 h at 37 °C and 5% CO_2_ content in humid atmosphere. Then PTMTC was washed out and cells were fixed (4% paraformaldehyde in PBS at 37 °C for 15 min, 0.2% Triton X-100 for 5 min) and stained with concanavalin A, Alexa Fluor 647 Conjugate (Con A, Molecular Probes, the working concentration: 100 µg/mL). Confocal imaging was carried out using the Zeiss LSM 780 system.

### In vivo Toxicity Testing

The experiment was performed according to the procedure OECD 420 [23], with permission obtained from Local Ethical Committee prior to the experiment. The tested compound was dissolved in PBS and administered to three female BALB/c mice intraperitoneally and to three female BALB/c mice intravenously (into the tail vein). The control animals received vehicle alone. The animals were observed for three days for symptoms of distress and pain according to generally accepted recommendations Carstens and Moberg [24]. Three days after administration of tested compound animals were sacrificed and blood samples were collected for further biochemical analyzes. The activities of the liver enzymes such as alanine aminotransferase (AlAT), asparagine aminotransferase (AsPAT), alkaline phosphatase (ALP), and lactate dehydrogenase (LDH) were measured using kits ordered from Pointe Scientific (Canton, MI USA) according to the manufacturer’s recommendations.

### Statistics

Statistical analysis was performed using GraphPad Prism™ 5.00 software (GraphPad Software, San Diego, USA). Results were expressed as means ± SD. Differences with a *p*-value < 0.05 were considered to be statistically significant.

## Results

### EPR Spectroscopy

**Fig. 1:**
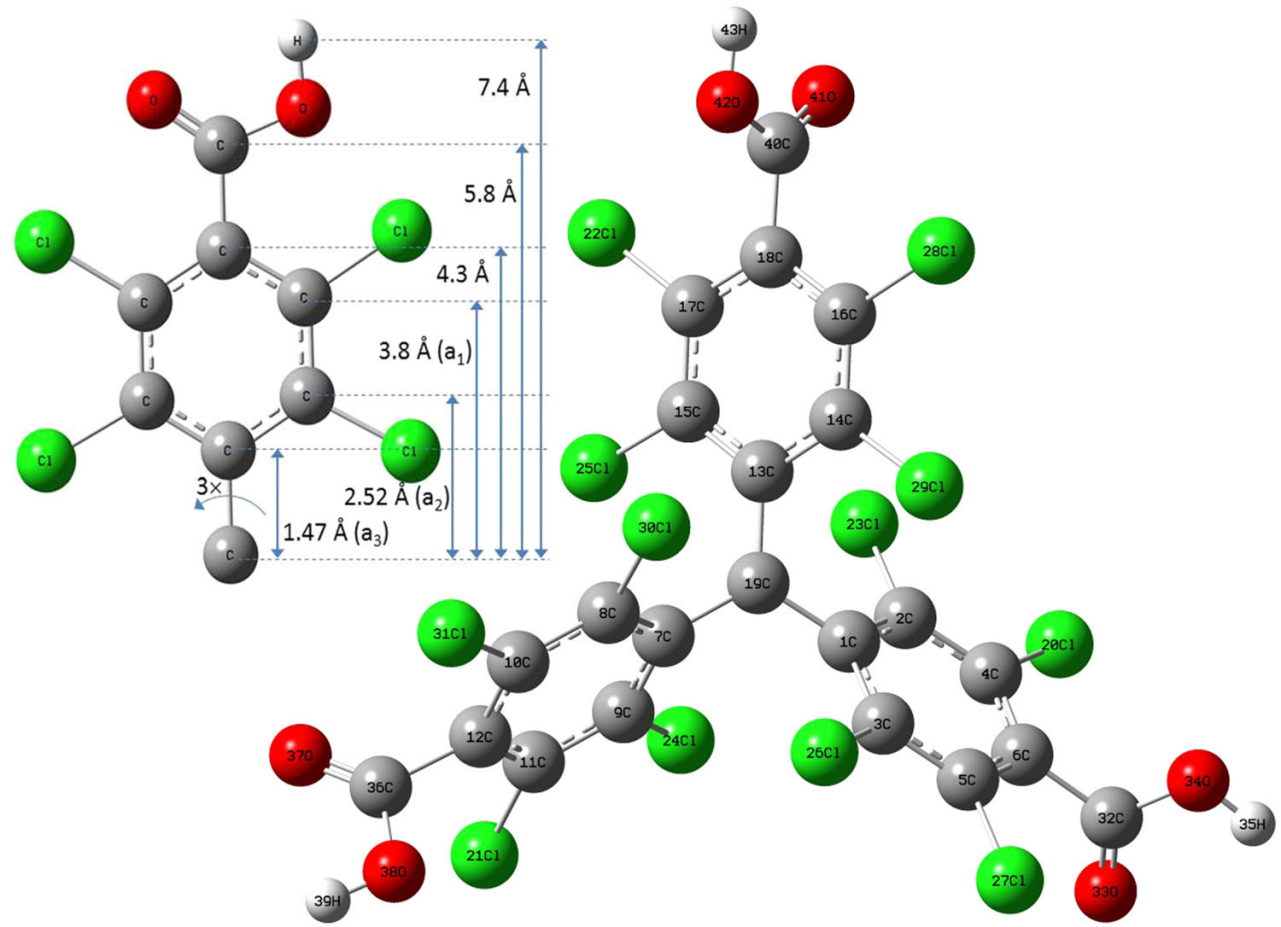
Molecular structure of PTMTC optimized in Gaussian visualized in GaussView (gray C, green Cl, red O, and white H) [30]. Inset shows radial distance through space from the center C atom with sources of isotropic shfs (color figure online)

**Fig. 2:**
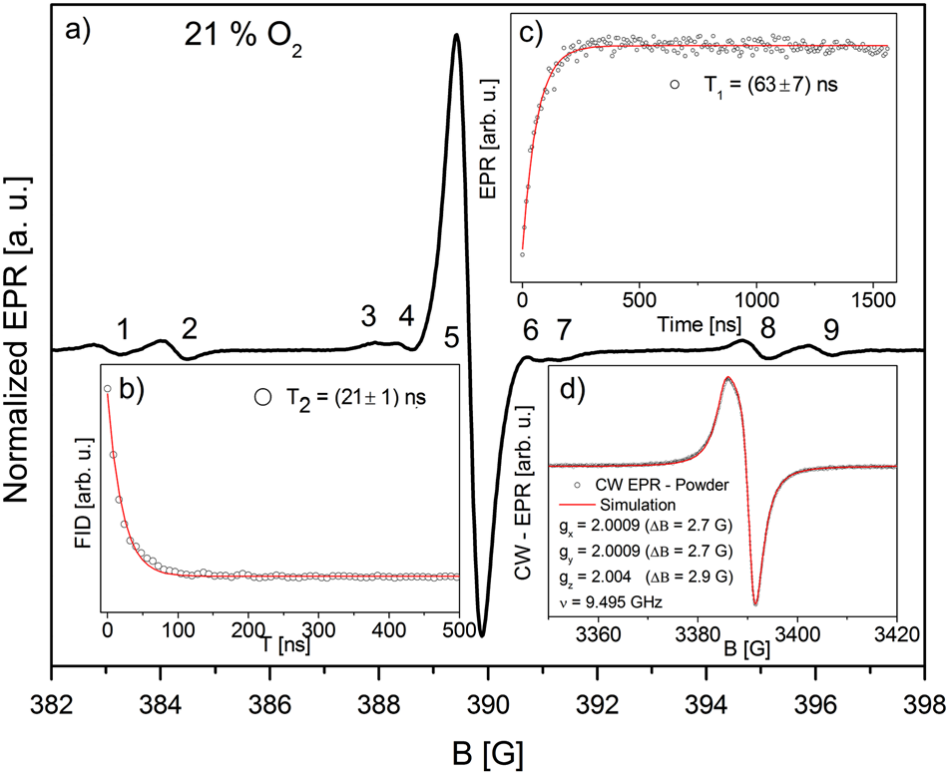
**a** EPR signal of PTMTC in PBS solution pH 7.4 (~18.5% O_2_) inset: **b** free induction decay (*T*_2_* = (21 ± 1) ns); **c** *T*_1_ = (63 ± 7) ns (from FID—saturation); **d** powder EPR line and simulation

The molecular structure was firstly optimized by DFT methods, to show the ground state molecular shape. The structure depicted in Fig. 1 was optimized using unrestricted ground state calculations with the b3lyp/6-311 + + g(d,p) basis set, without additional solvation model. The molecule exhibits C_3_ symmetry, where the normal to four central carbon atoms (1C, 7C, 13C, and 19C atoms) and the normal to the aryl group plane form an angle of around 45°. The normal to the –COOH plane (e.g., 43H, 42O, 41O, and 40C atoms) is rotated by 90° to the aryl plane (Fig. 2).

The EPR signal in solution (PBS pH 7.4) shows a radical *S* = ½ which interacts with multiple nuclei of carbon isotope ^13^C giving rise to multiple hyper- (a_4_ 13.08 G) and super-hyperfine structure transitions (shfs (a_3_, a_2_, and a_1_) Fig. 1; Table 1). Other nuclei having unpaired nuclear spins in the system are: ^1^H, ^35^Cl, and ^37^Cl, but their distance from the central atom or the abundance disqualifies them as the source of the super hyperfine coupling. An additional field sweep (100 G) with multiple accumulations was made and no further shfs were found.

**Table 1:** Isotropic hyper- and super hyperfine coupling constants (*ν*  = 1.09 GHz, 18.5% O_2_)

No.	1	2	3	4	5	6	7	8	9
B [G]	382.63	383.95	388.00	388.46	389.60	390.78	391.29	394.61	395.76
ΔB [G]	0.46	0.46	0.21	0.22	0.47	0.22	0.21	0.46	0.46
A [a. u.]	0.016	0.030	0.0022	0.0075	1	0.0075	0.0022	0.030	0.016
I [a. u.]	0.0034	0.0063	97 × 10^−6^	0.00036	0.22	0.00036	97 × 10^−6^	0.0063	0.0034
a_1_ [G]				2.32					
a_2_ [G]			3.29						
a_3_ [G]		10.66							
a_4_ [G]	13.08								

No. is the number of line in the Fig. 2, B is the field where the signal appears, ΔB is the peak–peak linewidth, *A* is the amplitude, *I* is the intensity from equation $I = A \cdot \Delta {B^2}$ [31], a_i_ is the isotropic Fermi coupling constants. For the assignment of couplings No. 1–9 to ^13^C atoms in PTMTC, see [32]

The EPR relaxation measurements were assessed at room temperature (23 °C). The spin–spin relaxation time *T*_2_* = (21 ± 1) ns and spin–lattice relaxation time *T*_1_ = (63 ± 7) ns were obtained from saturation recovery of FID. It was impossible to assess an electron spin echo, which means the line was homogeneously broadened. Short electron spin relaxation times do not encourage the use of this probe in pulse EPR imaging experiments [25, 26]. The powder spectrum shows an axial g-tensor with values *g*_*x*_ = *g*_*y*_ = 2.0009, and *g*_*z*_ = 2.004 which correspond to linewidths ΔB_*x*_ = 2.7 G, ΔB_*y*_ = 2.7 G, ΔB_*z*_ = 2.9 G.

### Sensitivity to Oxygen in Liquid Environment (PBS pH 7.4)

**Fig. 3:**
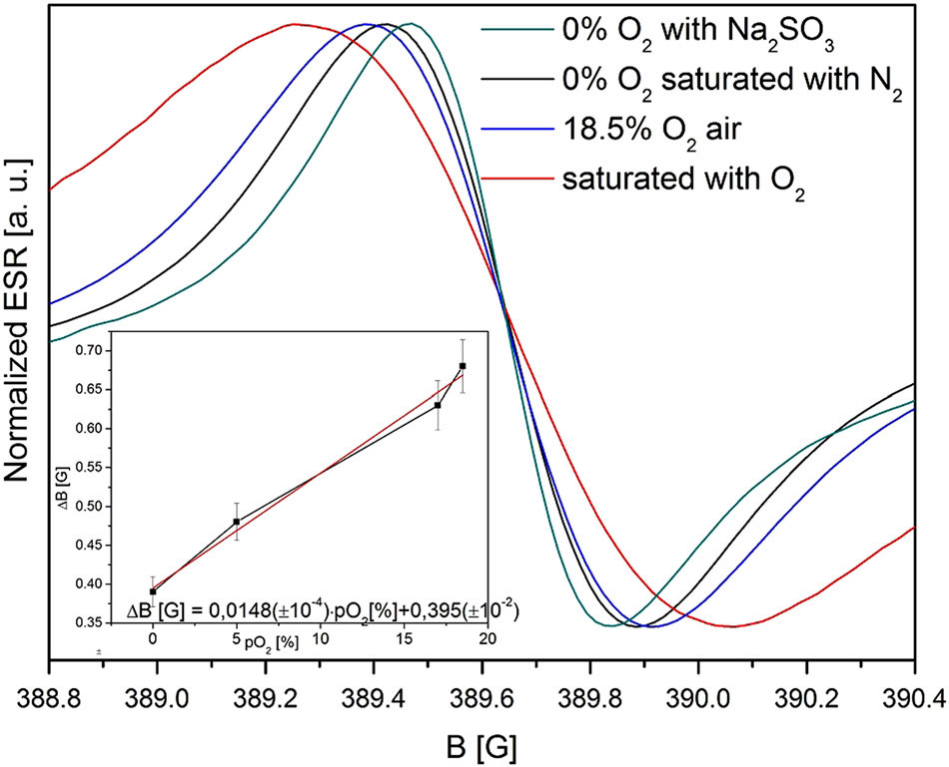
Central line broadening of the main EPR line component; (inset) linewidth vs. saturation with oxygen in PBS pH 7.4

The measurement of the EPR line width started after the PBS solution had been saturated with N_2_ gas. Oxygen content was out of the measurement range of the PreSense® oximeter. The lowest line width observed for the central signal was 0.39 G which in Fig. 3 was assigned to 0% pO_2_. After the first measurement free exchange with air through the surface was allowed. The measurements took 24 h in total reaching the initial value of 18.5% O_2_. The line width dependence vs. oxygenation can be approximated by the equation ΔB [G] = 0.0148 (±10^−4^) pO_2_[%] +0.395 (±10^−2^), in the range of 0–18.5% of pO_2_. Using chemical methods of deoxygenation (saturation with Na_2_SO_3_ powder), a line width of 0.37 G was achieved. The maximal line width observed after O_2_ saturation was 0.79 G (Fig. 3).

### Stability of the Radical in Different Solutions

The radical showed different temporal behavior dependent on solution used (Table 2). Maximum stability was observed in PBS solution where the signal amplitude decreased linearly with time and after 93.5 h ended at 97% of the initial amplitude. The radical signal in the second and third solution behaved similarly, decrease starting after 48 h and reaching 94% after 93.5 h. The strongest decrease, which started immediately, was observed in the full blood sample where the signal had completely disappeared after 72 h. The solution with only plasma did not show this behavior but it is important to mention that before measurement the plasma was stored at 5 °C for 4 days before use. Stronger temporal changes may occur with a fresh sample. No differences in the EPR signal were observed when EDTA as anticoagulant was used instead of heparin.

**Table 2:** Stability of the radical in different solutions

Time		0 (h)	24	48	72	93.5
Solvent		%	%	%	%	%
1	PBS buffer pH = 7.4	100	99	99	98	97
**2**	EMEM FBS 10% P/S 1% (0.75 mL) + PBS (0.25 mL)	100	100	100	94	94
3	McCoy’s FBS 10% P/S 1% (0.75 mL) + PBS (0.25 mL)	100	100	100	94	94
4	Rat blood + heparin (0.75 mL) + PBS (0.25 mL)	100	64	34	1.3	0
5	Blood plasma + heparin (0.75 mL) + PBS (0.25 mL)	100	100	100	100	100

**Fig. 4:**
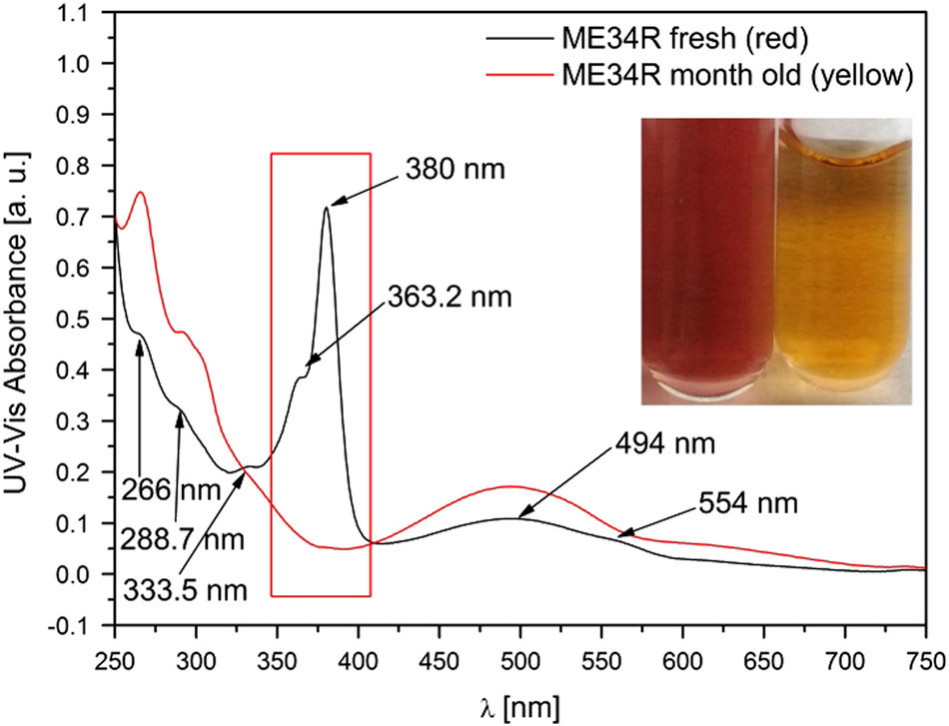
UV–Vis spectra of fresh and month old samples held under natural light conditions

The behavior of the sample in PBS solution was also tested after a longer storage at light conditions. The color of the PTMTC solution changed under the influence of light from red to yellow (Fig. 4). The difference was visible in the UV–Vis spectrum, where two strong lines at 363.2 and 380.0 nm vanished and the line at 494.0 nm became more dominant. This most likely was caused by the process of oxidative decarboxylation described by Decroos [27].

### Fluorescence Spectroscopy

**Fig. 5:**
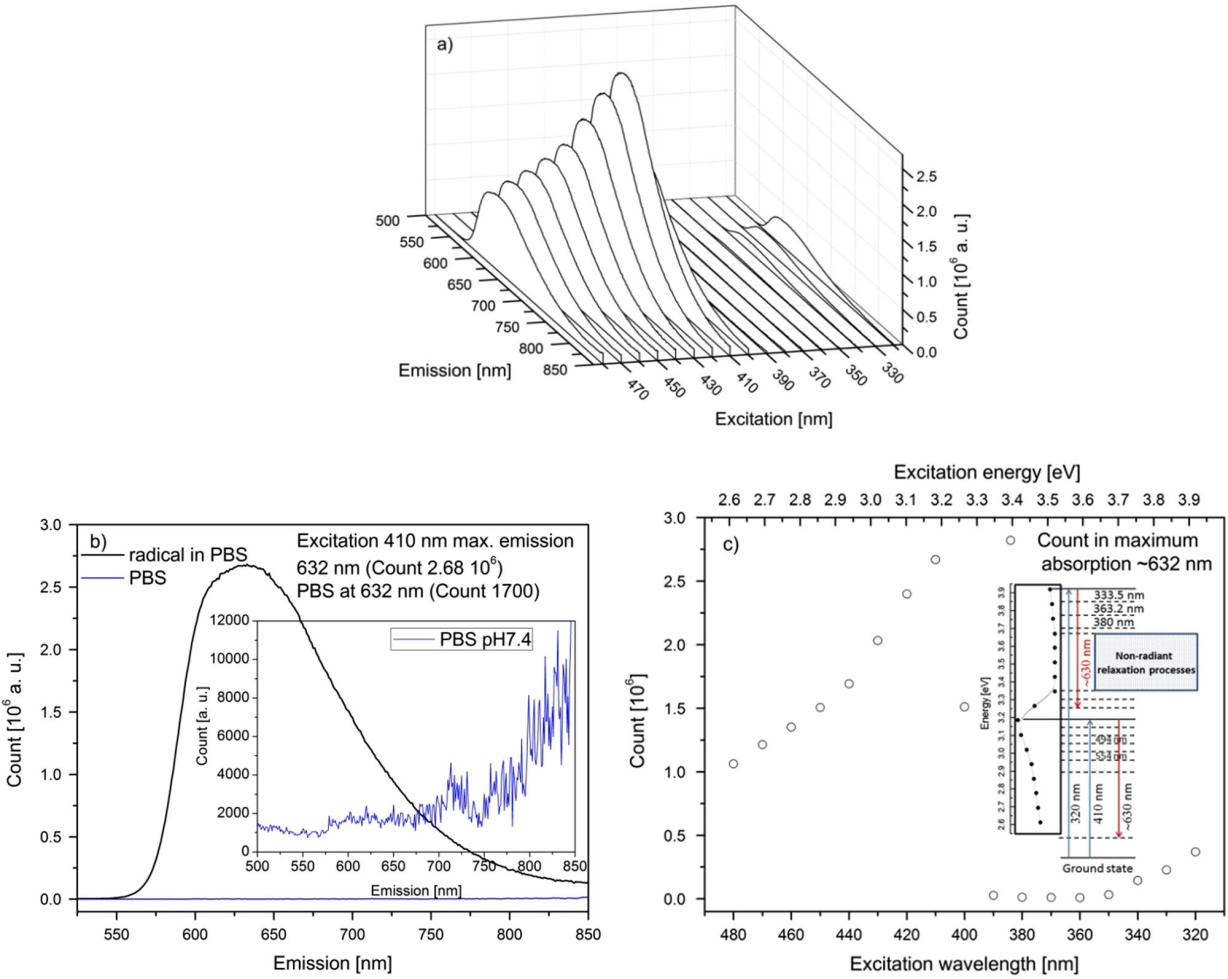
**a** Fluorescence vs. multiple excitation wavelengths; **b** The strongest fluorescence for the excitation at 410 nm (black) and PBS fluorescence (blue); inset shows fluorescence of PBS pH 7.4 buffer in larger scale; **c** Count in maximum absorption ~632 nm vs. excitation wavelength, inset shows proposed Jablonski diagram for this system (not in scale) (color figure online)

A PTMTC radical solution sample on excitation at 410 nm exhibited the strongest fluorescence emission peak with the maximum at 632 nm (linewidth at half height was 115 nm), with a Stokes shift of 1.06 eV (Fig. 5). Upon different excitation wavelength the fluorescence emission peak position did not change, however the intensity significantly varied. The pure PBS solution showed weak fluorescence, which vanished in the background. The ratio of counts at 632 nm for PTMTC radical solution and pure PBS sample was around 1574 (2.675 × 10^6^/1700). As an important index, the fluorescent quantum yield (QY) of PTMTC radical solution (14.2 µM) in PBS was determined by applying the following equation:

$${\rm{Q}}{{\rm{Y}}_{{\rm{sample}}}} = {\rm{Q}}{{\rm{Y}}_{{\rm{st}}}}\left( {\frac{{{I_{{\rm{sample}}}}}}{{{I_{{\rm{st}}}}}}} \right)\left( {\frac{{n_{{\rm{sample}}}^2}}{{n_{{\rm{st}}}^2}}} \right)\left( {\frac{{{A_{{\rm{st}}}}}}{{{A_{{\rm{sample}}}}}}} \right),$$

where QY is the quantum yield, *I* is the measured integrated emission at the excitation wavelength 488 nm, *A* is the absorbance at the excitation wavelength 488 nm, *n* the refractive index of the solvent. Rhodamine 6 G solution in ethanol served as standard (QY = 95%). In order to minimize the reabsorption effect, the absorbance of rhodamine standard and radical solution was kept below 0.1 at 488 nm excitation wavelengths. The fluorescence quantum yield of the PTMTC radical solution in PBS was calculated to be 5.7%. This is a very low QY when comparing to standard fluorescence dyes such as Rhodamine 6 G with QY of 94%, quinine sulfate (57.7%) and zinc phthalocyanine (30%). On the other hand, there are organic fluorescent dyes with similarly low QY e.g., chlorophyll *b* (7.4%). Also currently intensively investigated inorganic quantum dots do not reach high QY values, and yet they are successfully applied as fluorescent markers, e.g., dodecanethiol-capped CuInZn_*x*_S_2+*x*_ (25%) [28] or un-modified CdSeTe QDs (7.6%) [29].

Figure 5c shows the maximum fluorescence count vs. the excitation wavelength and energy. The most efficient radiant relaxation process appears for excitation energy of around 3.024 eV (410 nm). Red arrows indicate radiant relaxation process found in the fluorescence plot. Additional absorption levels are indicated from absorbance studies. As it seems non-radiant relaxation processes dominates in the range of 3.18 eV (390 nm) to 3.54 eV (350 nm), where the fluorescence count drops to a level of approx. 20,000. It rises again at the highest excitation energies used. Non-radiant relaxation processes could occur here in the form of vibrations (oscillations) of single or multiphonon transitions.

### Cytotoxicity

**Fig. 6:**
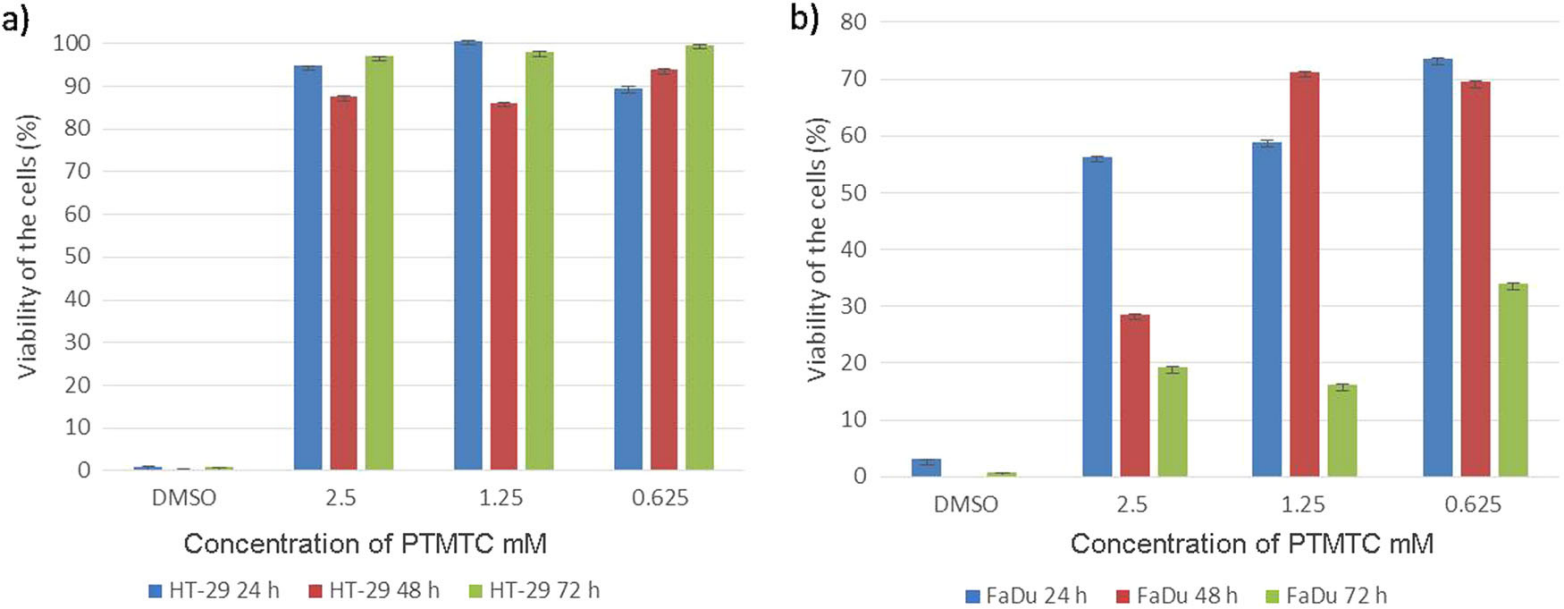
Viability of HT-29 (**a**) and FaDu (**b**) cell lines after 24, 48, and 72 h of incubation with PTMTC

After exposing HT-29 and FaDu cells to PTMTC, the cell viability was measured. It was observed that viability of HT-29 was high and stable, even at the highest dose (2.5 mM) and at the longest time of incubation (72 h) (Fig. 6a). The viability of FaDu cells after 24 h of incubation decreased with increasing PTMTC concentrations, and reached 73, 58, and 56% at concentrations of 0.625, 1.25, and 2.5 mM, respectively. After 48 and 72 h the same behavior of cellular response was observed (Fig. 6b).

### Cellular Uptake and Interaction with Luciferase–Luciferin Assay

The uptake and luciferase–luciferin assays were performed using the IVIS Spectrum system, which allows simultaneous measurement of both luminescence and fluorescence. The excitation and emission filters were selected experimentally to measure the fluorescence of PTMTC and exclude autofluorescence background of the cells itself. The experiment showed that PTMTC was not absorbed by cells and its presence had no impact on the fluorescence intensity produced by oxidation of luciferin in luciferase expressing LNCaP cells (data not shown).

### In vitro Confocal Studies

**Fig. 7:**
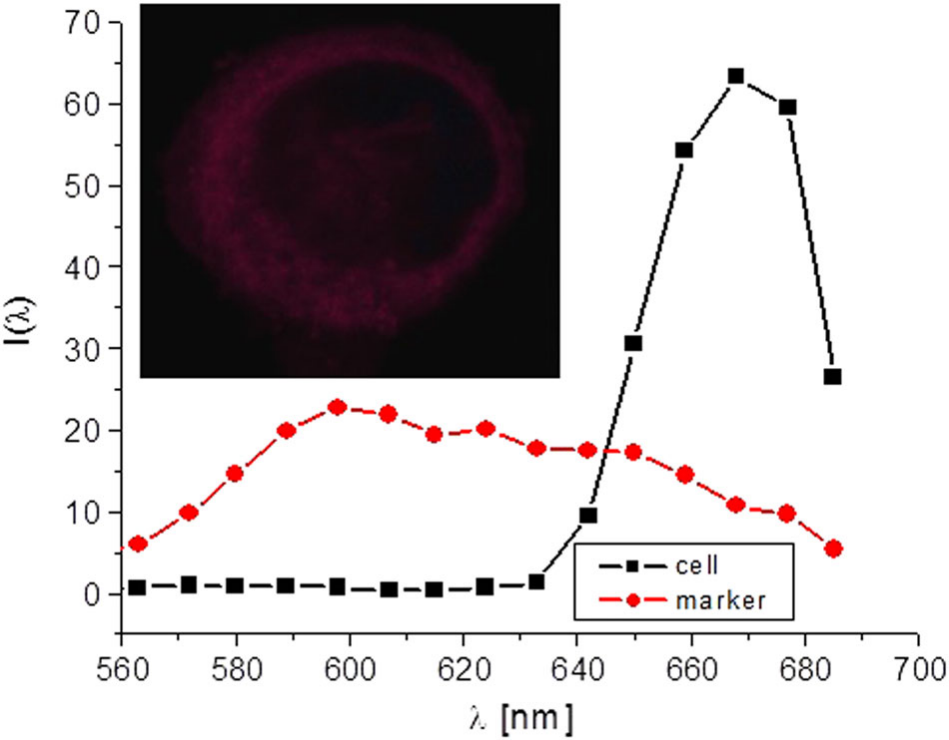
Emission spectra of the membrane of HT-29 cell incubated with PTMTC for 24 h and stained with concanavaline A (black squares) and of the PTMTC suspension in medium (red circles). Inset: LSM image of a HT-29 cell taken in the lambda mode

**Fig. 8:**
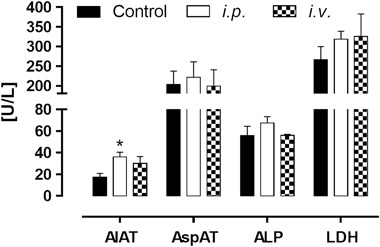
Effect of PTMTC on activity of AlAT, AspAT, ALP, and LDH 3 days after i.p. and i.v. administration of tested compound at dose 250 µg/kg BW. *Significantly different from control, *p* < 0.05

In order to unequivocally identify the fluorescent species, the images of the cell and of a PTMTC suspension were taken in the lambda mode with excitation at 405 nm, thus obtaining spectra in the range of 560–685 nm for every pixel in the images (Fig. 7). The black squares in Fig. 7 represent a typical spectrum of the cell stained with concanavaline A. Red circles refer to the spectrum of PTMTC suspension measured at the concentration used during the incubation (2.5 mM) and with the same imaging parameters. The use of the lambda mode allows for easy spectral separation of the signals from PTMTC and from concanavaline A. Fluorescence of PTMTC could be easily detected in the range of 560–620 nm where the contribution of concanavaline A is negligible. Fluorescence of PTMTC, if present, could be easily detected in the range of 560–620 nm where the contribution of concanavaline A emission is negligible. The main result of this measurement is that no such contribution of PTMTC fluorescence in the images of the cells incubated with this compound could be detected. (Fig. 8)

### In vivo Acute Toxicity Test

After administration of tested compound via both i.p. and i.v. routes, animals did not show any signs of pain or distress. Their behavior, motoric activity, food and water intake did not differ from the control group. After 3 days, the animals were sacrificed and their livers and kidneys were examined: no macroscopically visible lesions were found. In blood samples a significant increase of AlAT was measured in mice receiving PTMTC via intraperitoneal injection. The activity of other enzymes in both tested groups was slightly increased, however these changes were not significant.

## Conclusions

The EPR spectrum of PTMTC was recorded in an L-band spectrometer and analyzed. The signal of the radical displayed four different hyper and super-hyperfine coupling constants of different intensities which were assigned to interactions of the electron spin with ^13^C nuclei. Relaxation times of PTMTC in PBS, namely *T*_2_* of (21 ± 1) ns and *T*_1_ of (63 ± 7) ns, were obtained from saturation recovery of the FID. The linewidth sensitivity to O_2_ concentration was estimated. The temporal stability of the compound was tested in PBS, cell medium, full rat blood, and blood plasma. The molecule is stable in PBS solution, but decomposes immediately in blood. However, this does not prevent to use the PTMTC for in vivo studies. Therefore, the fluorescence properties of this marker were preliminarily investigated. The fluorescence quantum yield of the PTMTC radical solution in PBS was calculated to be 5.7%, which was rather low, but allows attempts to use this radical as a bimodal EPR/fluorescence imaging marker. In vitro time-dependent and dose-dependent cytotoxicity studies revealed the low toxicity of PTMTC against HT-29 and FaDu cell lines. Further tests showed that the compound was not absorbed by cancer cells and did not interfere with luciferin–luciferase based assays. PTMTC was also tested in vivo. It was administered to Balb/c mice at 250 mg/kg BW via both i.v. and i.p. administration. The radical caused only slight but statistically significant elevation of alanine aminotransferase in the i.p. group, while levels of AsPAT, ALP, and LDH remained unchanged. Mice receiving PTMTC after 3 days did not exhibit any signs of pain or distress nor did we notice any differences in food and water intake between control and test groups. The poor stability of PTMTC in blood indicates that it will not be stable in vivo for 3 days. However, such long-term stability is not needed for the planned combined EPR-fluoresence imaging, but it was important to show PTMTC did not interfere negatively with the host physiology.

In summary, PTMTC is a non-toxic oxygen sensor exhibiting fluorescent properties. Thereby, it is a perfect candidate for multimodal imaging with the prospect of quantitative information about oxygenation of the tissue (EPR) and distribution of the marker in the body (fluorescence).
